# Quantitative evaluation and obstacle factor diagnosis of drug regulatory capacity in China

**DOI:** 10.1371/journal.pone.0325924

**Published:** 2025-06-17

**Authors:** Mingming Zhai, Liwen Huang, Shijie Sun, Liying Cao, Xueqiong Yue, Yuanxia Hu

**Affiliations:** 1 School of Medical Devices, Shenyang Pharmaceutical University, Shenyang,; 2 School of Pharmaceutical Engineering, Shenyang Pharmaceutical University, Shenyang,; 3 School of Pharmacy, Shenyang Pharmaceutical University, Shenyang; Beijing University of Technology, CHINA

## Abstract

**Objective:**

To quantitatively evaluate the drug regulatory capacity in China, aiming to optimize the drug regulatory system, precisely enhance local regulatory effectiveness, and reduce regional regulatory disparities.

**Methods:**

Using the methods of literature research, expert interviews, investigation and analysis, the quantitative evaluation indicator system of supervision ability was established in all directions; the indicator data were collected and quantified; the indicator weight setting algorithm of the evaluation system was improved and the indicator weight was set by combining AHP and entropy method; the differences among eastern, central, and western provincial-level regions were analyzed by variance analysis; panel data were constructed for spatio-temporal evolution analysis; obstacle factor diagnosis model was used to analyze the obstacle factors.

**Results:**

The quantitative indicator system was constructed from five aspects: resource acquisition, function performance, learning and development, performance level and Internet application,and the relevant indicators of pharmacovigilance and risk response were analyzed at the national macro level. From the analysis of horizontal comparative variance, the comprehensive indicator and resource acquisition indicator of various provincial-level regions were significantly different(*P < 0.05*), while others were not significant. From the perspective of dynamic development, except for the performance level in 2022, all provincial-level regions were generally on the rise. From the perspective of obstacle factors, they were mainly in the aspects of learning development and functional performance. Regarding national pharmacovigilance and risk response, despite the synergistic development of all links ensuring drug safety and promoting industrial progress, new issues and challenges demand continuous attention and optimization of the regulatory system.

**Conclusion:**

There are regional differences in drug regulation in China. A drug regulation capacity improvement plan should be formulated in combination with the characteristics of the city itself and obstacle factors to achieve efficient and balanced development of drug regulation.

## 1. Introduction

Medicine is a special commodity used for the prevention, treatment and diagnosis of diseases. Whether the medicine can meet the requirements of safety and effectiveness is related not only to the safety of people's lives, but also to social stability and the credibility of the government. Therefore, governments all over the world attach great importance to the development and supervision of drugs. The strengthening of the drug regulatory system and the construction of drug regulatory capacity are the core driving force for accelerating the modernization of drug regulation and they are also the strategic basis for promoting the high-quality development of the pharmaceutical industry [1]. Since the reform and opening up, China’s drug regulatory system has been continuously improved, and the drug safety situation is generally stable. However, such incidents as “Qi Er Pharmaceutical Incident”, “Shandong Illegal Vaccine case” and “Changchun Changsheng Vaccine scandal” reflect that there are still shortcomings in China’s drug regulatory system and regulatory capacity [2]. In addition to strengthening whole-life-cycle drug supervision, the government should also stand on a higher level, think about the reasons behind the frequent occurrence of drug safety problems, learn from the experience of developed countries in Europe and the United States, focus on local drug regulation, and comprehensively improve its own regulatory capacity, so as to standardize the market order and protect public health [1].

China’s drug regulatory agencies mainly consist of the National Medical Products Administration (NMPA) and its subordinate local regulatory agencies. The NMPA is responsible for drug approval, supervision, and policy formulation across the country. Its functions include drug registration, supervision of production and circulation, drug safety monitoring, and reporting of adverse reactions. According to the Drug Administration Law, the responsibilities of the NMPA also include the supervision and management of drug clinical trials to ensure that drugs undergo strict safety and efficacy evaluations before being marketed [3]. Meanwhile, local drug regulatory departments are responsible for the specific supervision of drugs within their jurisdictions, including inspections of drug manufacturing enterprises, supervision of the drug market, and review of drug advertisements. Due to differences in local economic development levels, regulatory resource allocation, and policy implementation efforts, there are significant disparities in drug regulatory capabilities among different regions in China. Drug safety supervision in various regions of China is developing in a diversified way, so it is necessary to evaluate local drug regulatory capacity scientifically and effectively. Correct evaluation of drug regulation can not only improve the ability, analyze the performance and existing problems, but also encourage the supervision team to actively learn various professional knowledge, improve the overall professional quality, and comprehensively improve the quality of supervision [4–5].

The current research on drug regulatory capacity can be divided into two directions: theoretical research and empirical analysis. Theoretical analysis mainly studies and refines the theoretical connotation of government ability and drug regulatory capacity. The empirical analysis mainly studies and explores the construction of the evaluation system of supervision ability, including feasible operational indicators and data collection and analysis methods. Scholars have built evaluation systems based on different perspectives and ways, but few have carried out quantitative practical analysis [1,6–11]. According to the spirit of the “*Implementation opinions on comprehensively strengthening drug regulatory capacity building*” (Guo Ban Fa [2021] No. 16) document of the General Office of the State Council, the exploration of quantitative drug regulatory capacity evaluation model under the strategy of pharmaceutical power is the evaluation benchmark and future reference direction for the improvement of drug regulatory capacity [12]. Up to now, there is no comprehensive indicator system to establish the empirical measurement and quantitative analysis of the evaluation of drug regulatory capacity in various provincial-level regions across the country. This current situation makes it difficult for us to comprehensively and thoroughly understand the actual situation of the drug regulatory capabilities in various provincial-level regions. And only by conducting a comprehensive empirical quantitative analysis of the drug regulatory capabilities of various provincial-level regions can we intuitively see the advantages and shortcomings of each provincial-level region, and achieve accurate improvement of regulatory efficiency. The Global Benchmarking Tool (GBT) of the World Health Organization (WHO) is of great significance in the field of drug regulatory capacity assessment. It points out the direction for countries around the world to construct evaluation indicator systems for drug regulatory capacity. However, China has unique national conditions. Drug regulation in China faces special challenges such as large regional differences, a complex industrial structure, and a large population. As a universal international assessment tool, the GBT focuses more on the overall regulatory capacity at the national level and does not adequately consider the huge differences among different regions in China. For example, there are significant differences in economic development levels among different regions in China, which directly affects the investment in regulatory resources and the exertion of regulatory capabilities. At the same time, China’s huge population base and complex medical needs pose unique challenges to drug regulation in ensuring public drug safety. The GBT may not fully consider the diversity and particularity of weight setting in different countries and has difficulty adapting to China’s complex regulatory environment. Based on the above analysis, this paper carried out a quantitative comparative empirical analysis of the drug regulatory capabilities of various regions in China, guided by the GBT and in light of China’s actual situation. The aim is to lay a foundation for optimizing the construction of China’s drug regulatory system, precisely improving local regulatory efficiency, and narrowing regional regulatory differences. The specific research objectives are as follows:

(1) Construct a quantitative evaluation system: Extensively collect relevant domestic and foreign literature, conduct in-depth research on the Global Benchmarking Tool (GBT) of the World Health Organization (WHO), and combine it with the actual situation of drug regulation in China, so as to construct a comprehensive, scientific and China-specific quantitative evaluation indicator system for drug regulatory capacity. Meanwhile, improve the algorithm for setting indicator weights by combining the Analytic Hierarchy Process (AHP) with the entropy method, enabling the determination of indicator weights to take into account not only subjective factors such as expert experience but also reflect the objective information of data, thereby enhancing the scientificity and reliability of the evaluation system and accurately measuring the actual level of China’s drug regulatory capacity.
(2) Precisely analyze regional differences: Quantitatively evaluate the drug regulatory capabilities of provincial-level regions in the eastern, central, and western regions of China. Through methods such as variance analysis and spatio-temporal evolution analysis, accurately reveal the differences and change trends of drug regulatory capabilities among different regions. Not only pay attention to the static differences in various aspects of different regions, but also conduct a dynamic analysis of the panel data from 2018 to 2022 to observe the development trajectories of drug regulatory capabilities in different regions. Identify the advantages and disadvantages of each region in drug regulatory capabilities, provide data support for the targeted formulation of regional regulatory strategies, and promote complementary advantages and collaborative development among regions.
(3) Diagnose obstacle factors: Use the obstacle factor diagnosis model to deeply analyze the key obstacle factors that affect the improvement of drug regulatory capacity. Calculate the obstacle degree of each indicator and subsystem for the improvement of drug regulatory capacity. Through the analysis of data from 2018 to 2022, identify the main factors that hinder the improvement of drug regulatory capacity and explore the performance differences of these factors in different regions, providing a basis for formulating precise improvement measures.
(4) Provide decision-making suggestions: Based on the above research results, provide practical policy suggestions for optimizing China’s drug regulatory system. According to the actual situation and obstacle factors of each region, put forward targeted improvement plans to help improve local regulatory efficiency, narrow regional regulatory differences, promote the balanced development of drug regulatory capabilities across the country, and further ensure public drug safety and promote the healthy development of the pharmaceutical industry.

## 2. Methods

### 2.1 Indicator system construction

#### 2.1.1 Literature research.

By utilizing resources from the National Medical Products Administration (NMPA) website, academic databases (e.g., CNKI, PubMed, Web of Science), and institutional libraries, we comprehensively collected various types of materials related to drug regulatory capabilities, covering drug regulatory policies, regulations, industry trends, as well as research achievements such as peer-reviewed articles, monographs, and conference proceedings on drug regulation and regulatory capabilities. Through systematic analysis of these materials, we synthesized key theoretical frameworks and empirical findings to establish a foundation for the indicator system construction.

#### 2.1.2 Construction of the indicator system based on WHO’s GBT.

The Global Benchmarking Tool (GBT) developed by the World Health Organization (WHO) encompasses nine basic functions, including the Regulatory System (RS), Marketing Authorization (MA), Pharmacovigilance (VL), Market Control (MC), Licensing of Institutions (LI), etc., providing a comprehensive framework for evaluating the regulatory systems of various countries [13]. This tool has broad international applicability and aims to provide standardized assessment dimensions for national drug regulatory systems from a macro perspective. However, due to significant differences among countries in aspects such as politics, economy, society, and culture, it needs to be adjusted in combination with the actual situations of different countries when being applied specifically.

When constructing the quantitative evaluation indicator system for China’s drug regulatory capacity, we conducted in-depth research on the nine basic functions of the GBT. These functions widely cover key aspects such as licensing, registration, market control, regulatory inspections, laboratory testing, post-marketing supervision, pharmacovigilance, clinical trial supervision, risk management, promotion of innovation, and international cooperation, which provided important references for us to determine the direction of the indicator system. On this basis, we further combined China’s actual national conditions, the practical feasibility of data acquisition, and the booming development trend of the Internet to establish the evaluation indicator system.

China has a vast territory, and there is an imbalance in regional economic development. Different regions have significant differences in aspects such as the pharmaceutical industry structure, the distribution of regulatory resources, and medical needs, all of which have an important impact on drug regulatory capacity. The GBT is a universal international assessment tool that focuses more on the overall regulatory capacity at the national level and has difficulty fully reflecting the huge differences among different regions in China. Therefore, our indicator system fully considers these regional differences and determines the weight coefficients of each indicator based on the actual needs of China’s drug supervision. Furthermore, we use the obstacle factor diagnosis model to diagnose the development obstacle factors in different regions. Through quantitative analysis, we provide a basis for formulating targeted drug supervision strategies for each region, which will help to improve the overall drug supervision level in China.

Meanwhile, in terms of data acquisition, although information technology has been continuously advancing, some regions still face problems such as inconsistent data statistical calibers, untimely data updates, and difficulties in obtaining some data, which to some extent limit the selection of indicators and the integrity of data. The GBT is usually constructed based on a relatively complete and unified data environment, while our research needs to adopt appropriate indicator selection and data processing methods to ensure the effectiveness of quantitative analysis under such complex data conditions.

In addition, the rapid development of the Internet has brought new opportunities and challenges to drug supervision. The GBT mainly focuses on traditional regulatory functions and may not have fully considered the impact of the Internet on drug supervision. Our indicator system has specifically set up indicators related to Internet applications, emphasizing that regulatory departments need to use Internet technology to enhance their capabilities and levels in improving supervision efficiency, serving the public and enterprises, so as to better adapt to the development trend of drug supervision in the Internet era.

Taking into comprehensive consideration of the above multiple factors and following the principles of objectivity, feasibility, simplicity, and rationality, we finally decided to construct the indicator system from several aspects such as resource acquisition, function performance, performance level, learning and development, and Internet application, and conduct a national-level analysis of pharmacovigilance and risk management [1,6,11]. This not only follows the key points of the GBT but also is highly consistent with the concepts advocated by the GBT, such as ensuring that resources support regulatory functions, managing the full life cycle of drugs, focusing on drug quality and market control, continuously improving regulatory capabilities, adapting to new trends, fulfilling the pharmacovigilance function, and promoting the high-quality development of the pharmaceutical industry. It also fully considers the characteristics of China’s drug market, realizes scientific quantitative analysis, and lays a solid foundation for demonstrating the achievements of China’s drug supervision in the context of international regulatory convergence.

#### 2.1.3 Expert interview.

The final indicator is determined by the way of expert interview to ensure the accuracy and authority of the indicator. Seven experts with more than five years of work experience in the industry are selected from drug regulatory agencies, industry associations and other departments to carry out Delphi expert opinion consultation and determine the final evaluation indicator system. Further invite experts to score the importance of indicators as the basis for setting subjective weights. The importance of each indicator is measured on a five-component scale. The higher the importance of the indicator, the higher the score assigned [14]. The study employed an online survey methodology and strictly adhered to ethical guidelines outlined by the relevant institutional review board. The survey did not involve minors, and all data handling was conducted anonymously to ensure participant privacy. Prior to initiating the survey, clear and detailed explanations of the objectives were provided electronically, and digital written informed consent was obtained from all participants (Consent was obtained through WeChat, a widely used digital communication platform). Furthermore, the survey reiterated the purposes of the study and the usage of data to ensure participants were fully informed, thus safeguarding the scientific integrity, reasonableness, and ethical considerations of our research approach. The ethics committee approved our comprehensive consent process.

### 2.2 Weighted coefficient algorithm improvement for indicator system

#### 2.2.1 AHP.

Analytic Hierarchy Process (AHP) is a kind of research method which combines qualitative and quantitative methods to calculate decision weight for complex multi-objective problems. This method combines quantitative analysis with qualitative analysis, uses the experience of decision makers to judge the relative importance of the criteria to measure whether the goals can be achieved, reasonably gives the weight of each standard of each decision scheme, and uses the weights to calculate the order of pros and cons of each scheme [15]. This article determines the final indicators based on the results of interviews and investigations, and assigns weights to the indicators according to the scoring results of the respondents. The weight of each indicator for each subject is equal to the proportion of the importance score of that indicator to the total score of the importance scores of all indicators. Here, $w_{j}$ represents the indicator weight coefficient, and $d_{j}$ represents the importance score of each indicator.


$$w_{j}=\frac{d_{j}}{\sum_{j=1}^{m}d_{j}},(j=1,2,3,\cdots ,m)$$ (1)

#### 2.2.2 Entropy method.

In information theory, entropy is a measure of uncertainty or randomness, and the greater the uncertainty, the greater the entropy; The lower the uncertainty, the lower the entropy. The greater the uncertainty, the greater the randomness, the more discrete the data, the greater the information contains, and the smaller the coefficient tends to be when determining the weight.However, the entropy method only considers the dispersion degree of the data itself, and does not consider the information of the data in practical application. Suppose that there are n samples m indicators in the data, where the jth target of the i sample is represented. After the data is standardized, the specific weight calculation steps are as follows [16]:

a. Calculate the proportion of the i sample scheme of the j indicator in the indicator, and the formula is as follows:


$$P_{ij}=\;\frac{x_{ij}}{\sum_{i=1}^{n}x_{ij}}\;(j=1,2,3,\cdots ,m)$$ (2)

b. Determine the information entropy of each indicator


$$E_{j}=\;-\frac{1}{lnn}\sum_{i=1}^{n}P_{ij}\ln P_{ij}$$ (3)

c. Determine the weight of each indicator


$$A_{j}=\;\frac{1-E_{j}}{m-\sum E_{j}}(0\leq j\leq m)$$ (4)

#### 2.2.3 Improvement of the combination of AHP and entropy method.

Subjective weight setting is greatly affected by subjective consciousness, while objective weight setting depends on data, which may result in inconsistency with the actual situation. Therefore, this paper combines the two and adopts the subjective and objective combination weighting method to eliminate subjective bias and objective one-sidedness, so that the weight determined reflects both subjective and objective information, and can truly and completely reflect the actual situation of the impact of various indicators on drug regulatory capacity [17]. There are n indicators, $A_{j}$represents the coefficient calculated by entropy method for indicator j, $B_{j}$represents the coefficient calculated by AHP for indicator j, then the coefficient of the final indicator j is


$$W_{j}=\;\frac{A_{j}B_{j}}{\sum_{j=1}^{n}A_{j}B_{j}}$$ (5)

### 2.3 Analysis on the difference of drug regulation level in eastern, central and western regions

The economic development of the eastern provincial-level regions is fast, while the development of the central and western provincial-level regions is slow, and there is a large number of brain drain. Various indicators of drug regulatory ability in eastern, central and western provinces were analyzed by variance analysis to determine whether there were significant differences in drug regulatory ability among various provincial-level regions (*P < 0.05* was considered as significant differences).

### 2.4 Obstacle factor diagnosis model analysis

In order to further explore and identify the obstacle factors that restrict the improvement of drug regulatory capacity and provide decision-making reference for the targeted formulation of drug regulatory capacity improvement policies, the obstacle factor diagnosis model was introduced [18]. The obstacle factor diagnosis model determines the impact of each obstacle factor on the improvement of drug regulatory capacity through the obstacle degree. The calculation formula is as follows:


$$P_{ij}=\frac{(1-y_{ij})\times w_{j}}{\sum_{j=1}^{n}\left[(1-y_{ij})\times w_{j}\right]}\times 100\;\%$$ (6)

In the formula, $y_{ij}$ is the normalized value of the jth indicator of the i evaluation object, $w_{j}$ is the weight coefficient of the indicator, and $P_{ij}$ is the obstacle degree of the jth indicator for the i evaluation object to improve the comprehensive indicator of drug regulatory ability.

### 2.5 Data source and data processing

#### 2.5.1 Data sources.

The data in this study were derived from the annual report of drug statistics of the drug administration of all provincial-level regions in China from 2018 to 2022, the annual report of the government website, the final accounts of the drug administration departments and the statistical yearbooks of all provincial-level regions in China (considering the special nature of management and the availability of data, Hong Kong, Macao and Taiwan were not considered, and 31 provincial-level regions were included in the study). The regional informatization level algorithm is derived from literature [19]. During the data collection process, we developed strict data screening criteria to ensure the accuracy and reliability of the data. For example, for data with inconsistent statistical calibers, we made unified adjustments; for missing data, methods such as linear interpolation were used for supplementation. In addition, when analyzing the relevant indicators of pharmacovigilance, due to the need to understand the situation from a national macro perspective in the research, some data are sourced from national-level statistical materials, including but not limited to the data released by the National Adverse Drug Reaction Monitoring Center and the statistical information on the approval of new drug clinical trials by the National Medical Products Administration. These data support the macro-analysis of pharmacovigilance and risk response, and contribute to the overall assessment of China’s drug regulation in this area.

#### 2.5.2 Data processing.

The dimensions of each indicator are different, and different indicators have different influences on the evaluation of the subject. Among them, the indicators with positive effects are defined as positive indicators, and the indicators with negative effects are defined as negative indicators. In order to eliminate the differences among indicators, dimensionless processing was carried out for indicators before analysis, in which positive indicators were normalized and negative indicators were reverse-processed. The processing formula is as follows:

Normalization:


$$r=\frac{x-x_{\min}}{x_{\max}-x_{\min}}$$ (7)

Reverse:


$$\;r=\frac{x_{\max}-x}{x_{\max}-x_{\min}}$$ (8)

## 3. Empirical analysis

### 3.1 Application of indicator system and weight setting in empirical analysis

Set up the indicator system and determine the weights based on the methods in Sections 2.1 and 2.2. The specific details are as follows:

The resource acquisition indicator mainly measures the regional economic level in China and the human input in drug regulation. Per capita GDP and per capita fiscal revenue are important manifestations of regional economic strength. They directly affect the local government's ability to invest funds in drug regulation and are further related to aspects such as the purchase of regulatory equipment, technological updates, and personnel training. The regional informatization level reflects the comprehensive development degree of a region in terms of information infrastructure (such as information infrastructure, human resources, etc.) and application (such as Internet penetration, information consumption, etc.) [19]. It reflects the local Internet application ability, and in drug regulation, it will directly affect network utilization, such as influencing the transmission speed of regulatory information, the degree of data sharing, and the application effect of information technology in the regulatory process.

The function performance indicator reflects the actual implementation effectiveness of various functions of the local drug regulatory department in daily work. The number of administrative acceptance cases intuitively reflects the local drug regulatory department's handling ability and workload for various drug-related affairs and embodies its management efficiency in aspects such as market access. The number of approvals for the re-registration applications of domestic drugs reflects the continuous supervision intensity of marketed drugs, ensuring that drugs continue to meet quality standards throughout their life cycle. The relative value of the change in the number of registered practicing pharmacists refers to the change in the group of all practicing pharmacists registered in accordance with the law by the drug regulatory department. To some extent, it can reflect the guiding role of local drug regulation in the construction of a professional talent team and the development trend of the drug retail market. If the number of registered practicing pharmacists in a certain region shows a continuous growth trend, it indicates that the region has taken active and effective measures in attracting and cultivating pharmaceutical professionals, which will greatly improve the professional quality and safety guarantee level of drug retail services.

The performance level indicator reflects the overall quality status of drugs on the market and is a key indicator for measuring the actual effectiveness of regulatory work in ensuring drug quality. The pass rate of the production department reflects the quality control level at the source of drug production and reflects the supervision intensity of the regulatory department on production enterprises and the effectiveness of the production enterprises’ own quality management systems. The pass rate of the business department reflects the quality maintenance situation of drugs in the circulation process, including the management levels of warehousing, transportation, sales, and other links, and is an important link in ensuring the public's drug safety.

The learning and development indicator reflects the local attention and support for scientific research on drug regulation and is of crucial significance for promoting regulatory technology innovation, method improvement, and the research and solution of new problems. Scientific research investment can provide a solid financial guarantee for projects such as drug quality risk assessment and the research and development of new detection technologies, helping to improve the monitoring ability of regulatory departments on drug quality and safety. Training fees reflect the investment in updating the professional knowledge and improving the business capabilities of drug regulatory personnel. Through continuous training, regulatory personnel can better adapt to the constantly changing drug regulatory environment, master new regulations, policies, technical standards, and regulatory methods, and improve the scientific and effective nature of regulatory work.

The Internet application indicator evaluates the capabilities and levels of regulatory departments in using Internet technology to improve regulatory efficiency and serve the public and enterprises, providing strong support for promoting the modernization of drug regulation. The total website visits to some extent reflect the public's attention and demand for information related to drug regulation, and also embody the effect of the regulatory department in disseminating information through the Internet platform. A relatively high number of visits may indicate that the public pays great attention to drug safety issues, or the information released by the regulatory department has high practical value and attractiveness. The quantity and quality of released information reflect the degree of openness and publicity of regulatory departments on information such as drug regulatory policies, regulations, standards, and drug safety knowledge, which helps to improve the public's awareness and participation in drug regulatory work. The proportion of the number of government service items that can be handled entirely online measures the level of regulatory departments in using Internet technology to improve administrative efficiency and optimize service processes, providing more convenient and efficient services for enterprises and the public and promoting the informatization and intelligent development of drug regulatory work.

After determining the indicators, based on the improved algorithm combining the Analytic Hierarchy Process (AHP) and the entropy method, the influence coefficients of each indicator on the dependent variable are determined to calculate the six aspects of resource acquisition, function performance, performance level, learning and development, Internet application, and the comprehensive drug regulatory capacity indicator of each province and municipality. The comprehensive indicator is a comprehensive manifestation of various aspects of capabilities. The specific indicators and their weights are shown in Table 1. This table provides a clear and definite basis for the quantitative evaluation of China’s drug regulatory capacity, helps to comprehensively and objectively assess the current situation of drug regulatory capacity in various regions of China, discovers advantages and disadvantages, and provides strong support for further optimizing regulatory strategies and improving regulatory efficiency.

**Table 1:** Indicator system and indicator weights table for drug regulatory capacity.

Capacity	indicator		unit	weight	indicator nature
Comprehensive indicator of drug regulatory Comprehensive indicator of drug regulatory capacity	Resource acquisition capability (0.153)	Per capita GDP(I_1_)	Ten thousand yuan	0.281	Positive indicator
		Per capita fiscal revenue(I_2_)	Ten thousand yuan	0.379	Positive indicator
		Regional informatization level(I_3_)	\	0.340	Positive indicator
	Functional performance capacity (0.218)	Number of administrative acceptances(I_4_)	piece	0.562	Positive indicator
		The number of approved applications for re-registration of domestic drugs(I_5_)	piece	0.401	Positive indicator
		The relative value of change in the number of licensed pharmacists registered(I_6_)	\	0.038	Positive indicator
	Performance development level (0.289)	Qualified rate of drug sampling(I_7_)	%	0.152	Positive indicator
		Production department qualified rate(I_8_)	%	0.588	Positive indicator
		Operating department qualified rate(I_9_)	%	0.261	Positive indicator
	Learning and development ability(0.210)	Scientific research input(I_10_)	Ten thousand yuan	0.721	Positive indicator
		Training expense(I_11_)	Ten thousand yuan	0.279	Positive indicator
	Internet application(0.130)	Total site visits(I_12_)	number of times	0.330	Positive indicator
		Release information(I_13_)	number of times	0.580	Positive indicator
		The proportion of the number of government affairs that can be handled online in the whole process(I_14_)	%	0.091	Positive indicator

In addition, pharmacovigilance and risk response reflect the regulatory department's ability to monitor, evaluate, and respond to risks throughout the entire life cycle of drugs. However, in the actual research process, we have faced numerous difficulties, among which the problem of data acquisition is particularly prominent. At present, only data at the national level can be collected, and it is difficult to achieve horizontal comparisons among provinces and municipalities. Moreover, the data only includes the number of adverse drug reaction reports, the number of serious adverse drug reaction reports, the number of clinical trial approvals, and the pass rate.

The number of adverse drug reaction reports reflects the coverage and sensitivity of post-marketing safety monitoring of drugs. Timely and accurate reports are helpful for discovering potential drug safety problems. The number of serious adverse drug reactions intuitively reflects the frequency of serious hazards in the actual application of drugs and is the key to evaluating the potential risks of drugs. Its fluctuations affect the allocation of regulatory resources and policy adjustments and are of great significance for ensuring drug safety. The number of clinical trial approvals and the pass rate demonstrate the key efficacy of supervision from the access link at the front end of drug research and development. To some extent, the number of clinical trial approvals reflects the activity and innovation vitality of pharmaceutical research and development. A high number indicates that the regional pharmaceutical research and development industry is developing well and reflects the support of regulatory policies for innovation and the local research and development resource situation. The pass rate is a precise manifestation of the scientific review, risk control, and compliance with regulations by regulatory departments. A reasonable pass rate ensures the quality of clinical trial projects and balances public health and pharmaceutical innovation. Therefore, these four indicator data are selected, and descriptive statistical methods are used to analyze the overall national trend. Although there are limitations, it also provides a direction for future breakthroughs.

### 3.2 Analysis of regulation capacity of provincial-level regions

#### 3.2.1 Classification of eastern, central and western provincial-level regions.

The three economic belts refer to the economic region division method adopted in China in the 1980s. During the period of China’s seventh Five-Year Plan, the country was divided into three economic zones: eastern region, central region and western region. The specific classification results are shown in Table 2.

**Table 2:** Classification table of provincial-level regions.

Categories of provincial-level regions	Provincial-level regions name
Eastern region	Beijing, Tianjin, Hebei, Liaoning, Shanghai, Jiangsu, Zhejiang, Fujian, Shandong, Guangdong, Guangxi, Hainan
Central region	Shanxi, Mongolia, Jilin, Heilongjiang, Anhui, Jiangxi, Henan, Hubei, Hunan
Western region	Chongqing,Sichuan, Guizhou, Yunnan, Tibet, Shanxi, Gansu, Ningxia, Qinghai, Xinjiang

#### 3.2.2 Analysis of each provincial-level region’s indicators.

According to the established indicator system, the indicators of various aspects of drug regulatory capacity of various provincial-level regions in 2022 are calculated, and the specific results are shown in Table 3.

**Table 3:** Table of indicator situation of each provincial-level region in 2022.

Provincial-level region	Categories	Ranking	Composite indicator	Resource acquisition indicator	Functional performance indicator	Performance level indicator	Learning development indicator	Internet application indicator
Beijing	east	2	0.172	0.144	0.334	0.053	0.281	0.022
Tianjin	east	10	0.119	0.071	0.233	0.042	0.191	0.036
Hebei	east	26	0.080	0.022	0.159	0.021	0.138	0.052
Shanxi	central	16	0.111	0.032	0.220	0.012	0.208	0.083
Mongolia	central	18	0.104	0.044	0.223	0.016	0.206	0.008
Liaoning	east	7	0.139	0.027	0.269	0.051	0.218	0.122
Jilin	central	5	0.147	0.017	0.327	0.059	0.267	0.002
Heilongjiang	central	8	0.138	0.017	0.310	0.033	0.277	0.003
Shanghai	east	4	0.162	0.150	0.299	0.013	0.286	0.075
Jiangsu	east	1	0.204	0.071	0.379	0.150	0.228	0.147
Zhejiang	east	2	0.172	0.080	0.316	0.059	0.257	0.154
Anhui	central	6	0.141	0.022	0.303	0.063	0.240	0.026
Fujian	east	12	0.115	0.053	0.244	0.022	0.223	0.003
Jiangxi	central	13	0.114	0.021	0.250	0.044	0.206	0.004
Shandong	east	11	0.118	0.033	0.251	0.058	0.193	0.006
Henan	central	22	0.096	0.012	0.212	0.035	0.177	0.008
Hubei	central	25	0.091	0.028	0.193	0.031	0.162	0.010
Hunan	central	29	0.075	0.016	0.164	0.017	0.146	0.012
Guangdong	east	23	0.094	0.056	0.184	0.055	0.129	0.016
Guangxi	east	27	0.079	0.011	0.173	0.013	0.160	0.019
Hainan	east	21	0.098	0.028	0.211	0.013	0.198	0.022
Chongqing	west	14	0.113	0.027	0.245	0.009	0.236	0.026
Sichuan	west	17	0.106	0.025	0.211	0.096	0.115	0.032
Guizhou	west	15	0.112	0.008	0.246	0.009	0.237	0.038
Yunnan	west	19	0.102	0.006	0.222	0.009	0.213	0.045
Tibet	west	24	0.093	0.010	0.196	0.005	0.191	0.059
Shanxi	west	9	0.121	0.029	0.252	0.011	0.240	0.061
Gansu	west	20	0.100	0.004	0.213	0.016	0.197	0.056
Qinghai	west	28	0.077	0.013	0.159	0.006	0.153	0.050
Ningxia	west	30	0.059	0.021	0.115	0.006	0.109	0.044
Xinjiang	west	31	0.038	0.020	0.072	0.006	0.066	0.031
National average	/	/	0.113	0.036	0.232	0.033	0.198	0.041
Eastern average	/	/	0.132	0.063	0.261	0.048	0.212	0.055
Central average	/	/	0.109	0.022	0.236	0.031	0.205	0.019
Western average	/	/	0.092	0.016	0.193	0.017	0.176	0.044

As can be seen from Table 3, the composite indicators of Zhejiang, Jiangsu, Shanghai, Beijing and Jilin rank in the top five in 2022, while the composite indicators of Ningxia, Xinjiang, Qinghai, Guangxi and Hunan rank relatively low. The national average composite indicator is 0.113, with 13 provincial-level regions above average. Compared with the eastern region, the central and western regions have great disadvantage in the three indicators of resource acquisition, function performance and learning development. This may be caused by the relatively backward economic development, lower level of science and technology, more brain drain and other factors. The central and western provincial-level regions need to strengthen the preferential policies for talent introduction, and strengthen the development of high-tech industries to improve the large regional resource gap. Some eastern developed provincial-level regions, which are lower than the national average in the Internet application indicator, such as Beijing and Tianjin, can take local advantage to improve the comprehensive capacity of drug regulation.

#### 3.2.3 Indicator difference analysis of eastern, central and western regions.

According to the established indicator system and the indicator situation of each provincial-level region in 2022, the variance analysis is carried out for the eastern, central and western provincial-level regions, and the specific results are shown in Table 4.

**Table 4:** Analysis of variance results in the eastern, central, and western regions.

	Analysis of variance (mean ± standard deviation)			*F*	*p*
	Eastern regions(*n* = 12)	Central regions(*n* = 9)	Western regions(*n* = 10)		
Resource acquisition capability	0.06 ± 0.04	0.02 ± 0.01	0.02 ± 0.01	8.649	0.001**
Functional performance capacity	0.26 ± 0.06	0.24 ± 0.06	0.19 ± 0.06	3.309	0.051
Performance development level	0.05 ± 0.04	0.03 ± 0.02	0.02 ± 0.03	3.054	0.063
Learning and development ability	0.21 ± 0.05	0.20 ± 0.05	0.18 ± 0.06	1.395	0.265
Internet application	0.05 ± 0.06	0.02 ± 0.03	0.04 ± 0.01	2.296	0.119
Composite indicator	0.13 ± 0.04	0.11 ± 0.03	0.09 ± 0.03	4.509	0.020*

* *p* < 0.05 ** *p* < 0.01.

**Fig 1:**
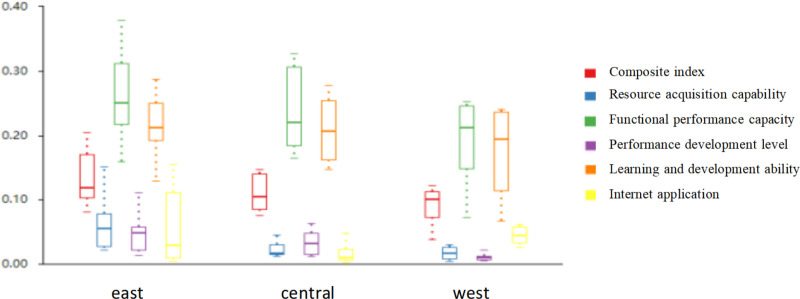
Comparison chart of different city category coefficients.

As can be seen from Table 4, the comprehensive indicator and resource acquisition indicator of various provincial-level regions have significant differences (p < 0.05). There is no significant difference in other indices. The comparison of coefficients of different provincial-level regions is shown in Fig 1.

It can be seen from the Fig 1 that provincial-level regions in the eastern region have certain advantages in drug regulation. Specifically, all indicators of the eastern provincial-level regions are the highest among the three regions and by comparison the learning and development indicator has the smallest gap with other regions. According to this, in order to expand the advantage, eastern region can increase scientific research investment to develop regional technology and also can strengthen training to enhance personnel's professional knowledge and ability, further to increase supervision ability.

The resource acquisition, functional performance, performance level and learning development indicator of the central provincial-level regions are higher than those of the western provincial-level regions and lower than those of the eastern provincial-level regions, and among all the indicators the Internet application indicator is prominent, which is the lowest. In order to improve this indicator, central provinces can strengthen the use of emerging technologies and online platforms. At the same time, other aspects should also be improved, such as relying on policies to promote regional economic development to provide a strong guarantee for the implementation of drug regulation, and regulatory departments should strengthen the administrative processing capacity and personnel training to enhance regulatory capacity.

Resource acquisition, functional performance, performance level and learning development of the western provincial-level regions are all in last place. compared with the corresponding indicators of the eastern provincial-level regions, the differences of each indicator are 0.04, 0.07, 0.03 and 0.03 respectively, and those of the central provincial-level regions are 0.00, 0.05, 0.01 and 0.02 respectively. We can first start with the performance of functions, and then improve the execution ability of performance level, increase the investment in science and technology and personnel training, to close the gap with the eastern and central provincial-level regions, and maintain the steady improvement of the Internet application indicator to strengthen the supervision ability of drugs.

#### 3.2.4 Dynamic development analysis.

**Fig 2:**
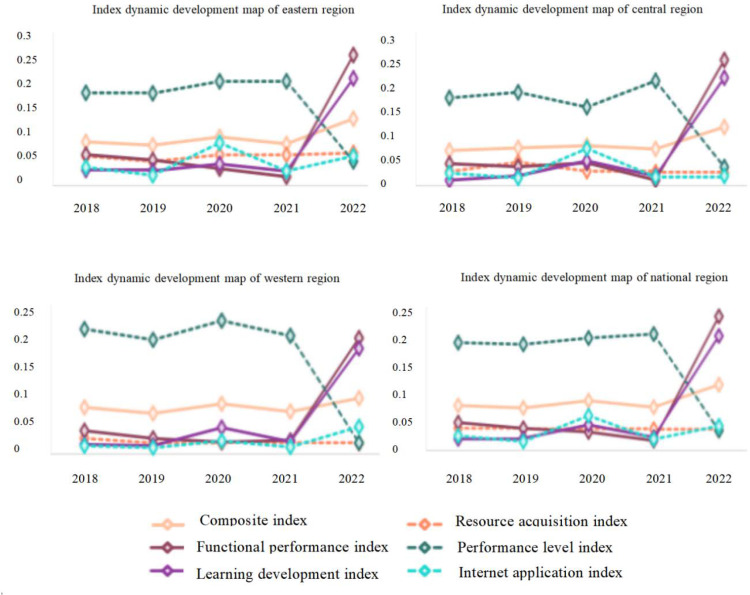
Dynamic development chart of indices of the eastern, central, western and national regions.

In order to understand the dynamic development of each provincial-level region and put forward the suggestions more scientifically according to the established indicator system, the indicators of each provincial-level region from 2018 to 2022 are calculated. The specific results are shown in Fig 2.

According to Fig 2, it can be seen that all indicators (except performance level) in the eastern region show an overall upward trend, the growth rate is significantly accelerated in 2021–2022, while the change of resource acquisition indicator is relatively gentle.

The learning development indicator, functional performance indicator and comprehensive indicator of the central and western regions generally have a good trend, and the Internet and resource access indicators have tended to be stable, with little change.

The reason for the sudden increase in 2020 in the indicators of each provincial-level region may be the sudden outbreak of the new coronavirus epidemic, the main work shifted online, and the information release of provincial and municipal drug administrations has increased, and the website page views have also increased. The regional performance level indicator drops sharply in 2022, which may be related to the increase of drug research companies and vaccine research and development related to the novel coronavirus outbreak. Now, after the epidemic has passed, all provincial-level regions should strengthen drug regulation, comprehensively improve the qualified rate of drugs, and use science and technology to develop a more standardized and efficient regulatory system. These indicators such as functional performance, resource access and Internet application show an upward trend in general and should continue to develop steadily. For indicators with a downward trend, strategies should be adjusted, advanced strategies of other provincial-level regions should be actively learned, and corresponding adjustments should be made according to their own characteristics, so as to improve the comprehensive ability of drug regulation.

#### 3.2.5 Obstacle factor diagnosis analysis.

On the basis of the comprehensive evaluation results of China’s drug regulatory ability, the obstacle factor diagnosis model was used to further explore the factors hindering the development of drug regulatory ability. According to the obstacle factor diagnosis model calculation method, the obstacle degree of each indicator and subsystem of the development of drug regulatory capacity during 2018–2022 is calculated, and the top two obstacle factors are selected according to the order of the obstacle degree from the largest to the smallest, as shown in Table 5.

**Table 5:** The main obstacle factors to the improvement of drug regulatory capacity in eastern, central and western provincial-level regions.

Regional	Project	2018		2019		2020		2021		2022	
Eastern region	Obstacle factor	I_11_	I_9_	I_11_	I_10_	I_8_	I_10_	I_10_	I_4_	I_10_	I_4_
	Obstacle degree	20.55%	15.22%	19.81%	17.53%	15.81%	15.38%	19.41%	18.13%	16.23%	15.72%
Central region	Obstacle factor	I_11_	I_9_	I_12_	I_10_	I_8_	I_10_	I_10_	I_4_	I_10_	I_12_
	Obstacle degree	19.97%	14.77%	17.91%	15.47%	15.93%	15.82%	14.89%	12.21%	20.84%	15.89%
Western region	Obstacle factor	I_11_	I_14_	I_11_	I_4_	I_8_	I_10_	I_10_	I_4_	I_10_	I_4_
	Obstacle degree	19.96%	15.53%	22.22%	14.90%	15.60%	15.46%	16.24%	11.96%	20.36%	16.51%

As can be seen from Table 5, the main factors affecting China’s drug regulatory capacity in the past five years are training cost (I_11_) and scientific research investment (I_10_), which reflects that the improvement of regulatory capacity is inseparable from learning and development. Especially in the aspect of scientific research investment, the obstacle degree shows an increasing trend year by year. The main obstacle factors in the eastern and central regions are basically the same. In 2018 and 2020, the main obstacle factors include the qualified rate of the operating department (I_9_) and the qualified rate of the production department (I_8_), respectively. The obstacle degree of the two obstacle factors has shown a downward trend in the past two years, indicating that all provincial-level regions have increased their attention to the supervision of the production and operation department, and the qualified rate is constantly improving. For the western region, economic aspects have a certain impact on drug regulation, and the number of professional regulators is also insufficient. There are still shortcomings in the Internet application (I_12_, I_14_) in the central and western regions, and the application of the Internet should be gradually increased to improve work efficiency. In the past two years, the main obstacle factors affecting the improvement of the national drug regulatory capacity are scientific research input (I_10_) and the number of administrative acceptance (I_4_), which indicates that we should pay more attention to learning development and functional performance, and constantly improve work efficiency while optimizing ourselves.

### 3.3 National pharmacovigilance and risk response

#### 3.3.1 Adverse drug reaction monitoring.

**Fig 3:**
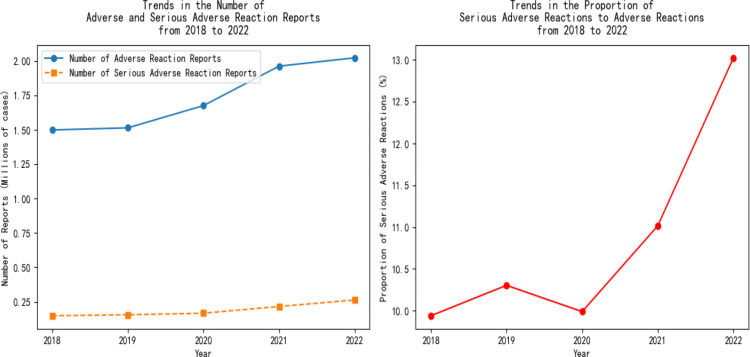
Trend chart of the number of adverse reaction reports.

The national adverse reaction report situation was sorted out. The adverse reaction situations from 2018 to 2022 are shown in Fig 3.

As can be seen from Fig 3, from 2018 to 2022, both the number of adverse reaction reports and the number of serious adverse reaction reports generally showed an upward trend. This reflects that significant progress has been made in China’s adverse drug reaction monitoring work. The monitoring network has become more and more complete, and the ability to collect data has become stronger, enabling the discovery of various reactions in drug use. It also reflects that the drug regulatory authorities attach importance to drug safety and have intensified monitoring efforts. The number of adverse reaction reports increased relatively steadily from 2018 to 2020, and the growth rate became larger from 2021 to 2022; the growth of the number of serious adverse reaction reports was more prominent from 2021 to 2022. There are several reasons behind this: First, the monitoring has been continuously deepened, and some potential serious risks that were not discovered in the past have been identified. For example, during the epidemic, long-term medication patients had extended medication time and increased combined drug use, which exposed originally hidden serious adverse reactions and led to more serious adverse reaction reports.Second, the epidemic has changed the disease spectrum. The conditions of some patients have become more complex and serious, requiring the use of potent drugs or a combination of multiple drugs for treatment. For instance, people with underlying diseases who contracted COVID-19 had an increased need for medication, and the risk of serious adverse reactions increased, resulting in a rise in the number of reports and their proportion. Third, the base number of drug use has increased. During and after the epidemic, people's demand for medication has increased. Whether it is for preventing and treating COVID-19 or dealing with other diseases, the scope and amount of medication have expanded, and the medication management of patients with chronic diseases has become more complex, all of which have increased the probability of adverse reactions. Looking at the proportion of the number of serious adverse reaction reports to the number of adverse reaction reports in each year, there were fluctuations from 2018 to 2022 but an overall upward trend. This means that among the monitored adverse reactions, the proportion of serious adverse reactions is increasing, and drug safety issues are more prominent. Higher attention needs to be paid to drug safety, and further analysis of factors such as the drug varieties, user populations, and administration routes that lead to serious adverse reactions is required to strengthen supervision and risk prevention and control more effectively.

#### 3.3.2 Approval of new drug clinical trials.

In terms of the approval of new drug clinical trials, in 2020, the number of applications for new drug clinical trials was 1,099, the number of approvals was 879, and the approval pass rate was 79.98%. At that time, the policies such as the reform of the national evaluation and approval system and the reform of clinical trial management had initially achieved results, stimulating the enthusiasm of enterprises to apply. However, affected by the initial stage of the epidemic, the response measures of all parties were still being explored and improved, and there were lags and uncertainties in new drug research and development and clinical trials, resulting in a relatively small number of applications.

In 2021, the number of applications for new drug clinical trials increased significantly to 1,830, with 1,483 approvals and a pass rate of 81.04%. On the one hand, the National Healthcare Security Administration carried out adjustments to the medical insurance drug catalog, encouraging pharmaceutical enterprises to increase their R & D investment in fields such as drugs for tumors and chronic diseases, and promoting relevant clinical trial applications. On the other hand, the support policies for the access of innovative drugs to medical insurance prompted enterprises to actively carry out R & D. In addition, the optimization of the evaluation and approval process created a favorable environment for new drug clinical trials, increasing the number of approvals and the pass rate. At the same time, the continuous epidemic prompted the pharmaceutical industry to conduct in-depth research and development of epidemic-related drugs, and the delayed release of some patients’ medical treatment needs and changes in their conditions gave rise to new drug demands, prompting pharmaceutical enterprises to intensify their R & D efforts.

In 2022, the number of applications for new drug clinical trials was 1,829, basically the same as that in 2021, while the number of approvals rose to 1,579, and the pass rate reached 86.33%. The continuous policy support stabilized the R & D expectations of enterprises, and the optimization of the evaluation and approval process improved efficiency. After the epidemic, patients’ conditions were complex, and the demand for new drugs was urgent. Pharmaceutical enterprises accelerated their R & D; the public and medical institutions paid more attention to the safety and effectiveness of drugs. The regulatory authorities carried out strict and meticulous supervision to ensure the quality of trials, improve the approval pass rate, and strengthen risk monitoring.

Overall, from 2020 to 2022, the number of applications and approvals for new drug clinical trials generally showed an upward trend, reflecting the active new drug research and development in China and the continuous increase in the support of regulatory authorities, promoting more new drugs to enter the clinical trial stage. The annual increase in the approval pass rate indicates that the quality of new drug research and development and the standardization and scientific nature of the application materials in China have gradually improved, and more applications that meet the regulatory requirements have been approved. It also reflects that the drug evaluation institutions have achieved remarkable results in optimizing the approval process and improving their professional evaluation capabilities, effectively promoting the new drug research and development process.

## 4. Conclusions and suggestions

### 4.1 Conclusions

In terms of methodology, this study is based on the GBT of the WHO. It closely integrates with the actual situation of drug regulation in China and comprehensively applies literature research and expert interviews to construct a quantitative indicator system covering five aspects: resource acquisition, functional performance, learning and development, performance level, and Internet application. When determining the indicator weights, the AHP and the entropy method are integrated to make the weight determination more scientific and reasonable. With the help of this system, in-depth analysis of regional differences has been carried out, the factors hindering the development of drug regulatory capacity have been diagnosed, the impacts of each indicator have been analyzed, and the situations of pharmacovigilance and risk response have also been analyzed from the national macro perspective.

Compared with the GBT, the analytical method constructed in this study is more in line with China’s national conditions. It can clearly show the advantages and disadvantages of the drug regulatory capabilities of each provincial-level region, providing strong support for precisely improving regulatory capabilities. Although limited by data acquisition, the indicator system has certain limitations. However, quantitative analysis and obstacle factor diagnosis still represent valuable analysis directions. In the future, the indicator system will be continuously optimized to provide a more reliable reference for research and practice in the field of drug regulation.

In terms of empirical results, based on the established indicator system, the indicators of each provincial-level region from 2018 to 2022 were calculated. From the horizontal comparison between provincial-level regions, there are regional differences in drug regulation capacity in our country. Zhejiang, Beijing and Shanghai’s comprehensive indicator ranking is relatively stable and high, but the development of the Internet application indicator is not stable.Therefore, Internet application, smart supervision should be the development focus of these provincial-level regions. Qinghai, Ningxia and Xinjiang all rank lower in the composite indicator in these five years, especially in terms of performance level. Such provincial-level regions should learn more from domestic and foreign experience, strengthen supervision, optimize supervision resources, and improve supervision programs. From the perspective of difference significance, there are significant differences in the comprehensive indicator of drug regulatory ability and resource acquisition ability indicator of eastern and central provincial-level regions, and there are also differences in other aspects, but the differences are not significant. From the perspective of their own dynamic comparison, in addition to the performance level of all provincial-level regions in 2022, the rest are generally on the rise, which also shows that with the economic and social development, the drug regulatory capacity of each provincial-level region in China is also gradually improving. The regional performance level indicator drops sharply in 2022, which may be related to the increase of drug research companies and vaccine research and development related to the novel coronavirus outbreak. All provincial-level regions should further strengthen the control of drug quality and the response to emergencies. Judging from the diagnosis of obstacle factors, the obstacle factors affecting the improvement of drug regulatory ability are mainly in the aspects of learning development and functional performance. From the perspective of national pharmacovigilance and risk response, all links of pharmacovigilance and risk response are developing in a coordinated manner, laying a solid foundation for ensuring drug safety and promoting the progress of the pharmaceutical industry. However, it is still necessary to continuously pay attention to new problems and challenges and constantly optimize and improve the regulatory system.

Overall, the quantitative indicator system constructed in this paper is more suitable for evaluating the development of drug regulatory capacity in each provincial-level region in China. There are regional differences in China’s drug regulatory capacity, and the drug regulatory capacity of each provincial-level region is also improving year by year. More attention should be paid to learning and development, functional performance, and Internet application. At the same time, each provincial-level region can formulate targeted improvement strategies based on its own characteristics and the diagnosis results of obstacle factors, so as to gradually narrow the regulatory differences among regions, promote the balanced and efficient development of drug regulatory capacity across the country, effectively guarantee public drug safety, and promote the healthy and sustainable development of the pharmaceutical industry.

### 4.2 Suggestions for improving drug regulatory capacity

Based on the above analysis results, the following specific measures are proposed to enhance China’s drug regulatory capabilities, aiming to achieve the comprehensive improvement and balanced development of drug regulatory capabilities.

(1) Promote the structural matching of regulatory resources and regulatory powers

In terms of resource acquisition, we should promote the structural matching of regulatory resources and regulatory powers.The Implementation Opinions of The General Office of the State Council on Comprehensively Strengthening the Capacity Building of drug regulation requires city and county market supervision departments to strengthen the allocation of drug regulation law enforcement forces in the comprehensive law enforcement teams to ensure that they have professional supervision personnel, funds and equipment that match the regulatory powers [2]. All provincial-level regions should establish clear regulatory objectives, innovate regulatory methods, optimize supervision and inspection methods, strengthen the resource allocation of local drug regulatory agencies at all levels in a targeted manner, and ensure that there is professional supervision, financial supervision, conditional supervision, and efficient supervision.

(2) Build a team of professional regulatory talents and a collaborative and efficient local drug regulatory system

In terms of functional performance and learning development, we should increase the investment in training and scientific research, attract high-level talents, improve the talent training system, and then build a professional regulatory talent team and a collaborative and efficient local drug regulatory system. Regulatory talent team directly affects work efficiency and results, and it is the key to functional performance. On the one hand, the regulatory talent team should be professional, keep learning, and keep up with the development of the times; On the other hand, it is also necessary to have a positive working attitude, change the status quo that drug regulation work is mostly centered on “no accidents”, and establish a professional belief of “ensuring public drug safety, protecting and promoting public health” [20]. At the same time, it is necessary to improve the departmental cooperation mechanism and information sharing mechanism [21], and strengthen the cooperation and exchange among various departments to promote efficiency through coordination.

(3) Improve the supervision mechanism of the whole life cycle of drugs, and vigorously develop smart supervision

From the aspect of performance level and Internet application, the supervision mechanism of the whole life cycle of drugs should be improved, and the supervision performance level should be improved through the innovation of supervision methods and the optimization of supervision and inspection methods. Pay attention to prior supervision, strengthen the dynamic supervision during and after the event [22], and effectively use social supervision [23]. Promote the progress and innovation of regulatory technology, strengthen the construction of drug regulatory technology support system, vigorously develop smart regulation, solve new problems in regulation through the learning and application of new technologies and new means such as big data, artificial intelligence and machine learning [24], and improve regulatory efficiency in an all-round way.

(4) Strengthen inter-regional collaboration and promote the coordinated development of supervision

To narrow the differences in regional supervision, inter-regional collaboration should be strengthened. The convergence of supervision policies and standards should be promoted. A unified national framework should be formulated and regions should be allowed to refine it appropriately. A coordination mechanism should be established to ensure consistency, such as unifying the requirements for drug registration application materials. A regional integrated intelligent supervision system should be constructed. Investment should be increased with a focus on the central and western regions. Unified data standards should be established to achieve information sharing. For instance, it is advisable to establish a national drug traceability platform to facilitate data sharing and analysis among regions. Developed regions should help underdeveloped regions improve their technological levels. Inter-regional cooperation and resource sharing should be strengthened. A supervision cooperation alliance should be established to conduct joint law enforcement and information sharing. Cooperation in talent cultivation should be enhanced to promote the flow of talents. An inter-regional difference assessment and coordination mechanism should be established to regularly assess the causes and formulate assistance plans. A permanent institution should be set up to be responsible for daily work and report progress. The interdependence among regions, the convergence of supervision, intelligent supervision, efficient coordination, and information sharing work together synergistically. Integrating resources to form a joint force will help improve the overall supervision efficiency and ensure the public's drug safety. The collaboration mechanism should be continuously optimized to meet the development needs and promote the progress of the drug regulation cause.

(5) Improve the pharmacovigilance system and enhance the precision of risk prevention and control

The pharmacovigilance system should be improved to enhance the precision of risk prevention and control in all aspects. On the one hand, it is necessary to integrate resources from all parties to build a national-level big data platform for pharmacovigilance, which can gather multi-source information including adverse drug reaction monitoring data, clinical trial data, and post-marketing monitoring data of drugs. Data mining and machine learning algorithms should be used to deeply analyze the correlations among the data, accurately identify potential risk signals, and achieve early warning and dynamic monitoring of drug risks. On the other hand, the construction of a professional team for drug risk assessment should be strengthened. Experts in multiple fields such as pharmacy, medicine, epidemiology, and statistics should be recruited. Based on international advanced standards and methods, the collected risk information should be scientifically and rigorously evaluated and graded to provide a decision-making basis for subsequent precise prevention and control. For high-risk drugs, personalized risk management plans should be formulated to clarify the supervision priorities and response strategies, ensuring that limited supervision resources are accurately allocated to key areas.

The above suggestions for improving drug regulatory capacity are put forward from various dimensions and in terms of narrowing regional differences. During the implementation process, each provincial-level region needs to closely combine its own actual situation, conduct in-depth analysis and focus on improving the diagnosed obstacle factor problems, so as to gradually achieve the steady improvement of drug regulatory capacity and reach the goals of more efficient, precise and coordinated drug regulation.

## 5. Research limitations and prospects

### 5.1 Research limitations

This study is dedicated to constructing a quantitative evaluation indicator system for China’s drug regulatory capacity and conducting in-depth analysis. During the research process, many valuable findings have been achieved, but inevitably, some limitations have been encountered. In terms of data acquisition, severe constraints have been faced. Some key data, such as pharmacovigilance risk data and the effect data of local special operations, are difficult to obtain, resulting in insufficient coverage of indicators. For example, in aspects such as the in-depth effect of Internet application and the efficiency of cross-departmental coordinated supervision, effective evaluation indicators are lacking, making it impossible to comprehensively and accurately analyze the actual impact of these factors on drug regulatory capacity. Meanwhile, the timeliness of the data lags behind, unable to reflect the impact of new policies and new technologies on regulatory capacity in a timely manner. Moreover, the sample scope does not include Hong Kong, Macao, and Taiwan regions, limiting the comprehensiveness of the research results. All these aspects need to be further improved and refined in the future.

### 5.2 Research prospects

Despite the above limitations, the quantitative evaluation indicator system constructed in this study still provides a valuable reference basis for the field of drug regulation. From the perspective of research methods, we have improved the weighted coefficient algorithm of the evaluation system. By innovatively combining the Analytic Hierarchy Process (AHP) and the entropy method, the scientificity and rationality of indicator weight setting have been effectively enhanced, thus achieving a more accurate quantitative evaluation of the drug regulatory capacity of each province and municipality. At the same time, the obstacle factor diagnosis model has been used to conduct an in-depth analysis of the obstacle factors affecting the improvement of drug regulatory capacity, accurately identifying the key restrictive factors and providing a clear direction for subsequent improvements. In addition, by constructing panel data and conducting spatio-temporal evolution analysis, a detailed comparison of the regulatory capacity performance of different provinces and municipalities in different time periods has been made, clearly presenting the dynamic change trend of regulatory capacity and providing strong support for an in-depth understanding of regional differences. At the research content level, we have systematically sorted out the performance of each province and municipality in key aspects such as resource acquisition, function performance, learning and development, performance level, and Internet application, and identified the advantages and disadvantages of different regions in these dimensions. This has laid a solid foundation for precisely improving local regulatory efficiency and targeted reduction of regional regulatory differences. Meanwhile, in the analysis of pharmacovigilance and risk response, the development trend and challenges faced by China in this important field have been deeply revealed. For example, although progress has been made in adverse drug reaction monitoring work, attention still needs to be paid to the increasing proportion of serious adverse reactions. The approval of new drug clinical trials has been continuously optimized under the impetus of policies, but still faces some uncertainties. These have provided key directions and important bases for further optimizing the regulatory system and strengthening risk prevention and control.

In the future, on the one hand, the indicator system should be continuously optimized. Emerging elements such as drug innovation incentives and the flexibility of regulatory policies should be incorporated, and indicators such as scientific research investment should be further refined to more accurately and comprehensively reflect regulatory capacity. On the other hand, big data technology should be utilized to mine diversified data, advanced algorithms should be applied to conduct in-depth analysis, research on regional differences should be strengthened, the coordinated development model should be actively explored, and extensive international comparisons and learnings should be carried out to continuously promote the improvement of China’s drug regulatory level and better ensure public drug safety.

## Supporting information

S1 File Raw_Data_2018.xls – Raw Data for Statistical Analysis (2018). (XLS)

S2 File Raw_Data_2019.xls – Raw Data for Statistical Analysis (2019). (XLS)

S3 File Raw_Data_2020.xls – Raw Data for Statistical Analysis (2020). (XLS)

S4 File Raw_Data_2021.xls – Raw Data for Statistical Analysis (2021). (XLS)

S5 File Raw_Data_2022.xls – Raw Data for Statistical Analysis (2022). (XLS)

S6 File Subjective_Weight_Calculation.xls – Raw Data for Subjective Weight Calculation. (XLS)
